# A Pilot EEG Study on the Acute Neurophysiological Effects of Single-Dose Astragaloside IV in Healthy Young Adults

**DOI:** 10.3390/nu17152425

**Published:** 2025-07-24

**Authors:** Aynur Müdüroğlu Kırmızıbekmez, Mustafa Yasir Özdemir, Alparslan Önder, Ceren Çatı, İhsan Kara

**Affiliations:** 1 Department of Basic Science, İstanbul Nisantasi University, Istanbul 34400, Türkiye; 2 SANKARA Brain and Biotechnology Research Center, Technology Park, Istanbul University Cerrahpasa, Istanbul 34320, Türkiye; musyasoz@gmail.com (M.Y.Ö.); alparslanonder@outlook.com (A.Ö.); sankaraenator@gmail.com (İ.K.); 3 Department of Biomedical Technologies, Izmir Katip Celebi University, Izmir 35620, Türkiye; 4 Göttingen Graduate Center for Neurosciences, Biophysics, and Molecular Biosciences, Georg August University of Göttingen, 37077 Göttingen, Germany; 5 Department of Biology, Faculty of Science, Istanbul University, Istanbul 34134, Türkiye; ceren.cati@ogr.iu.edu.tr

**Keywords:** Astragaloside IV, astragalus, EEG, brain oscillations, alpha/beta ratio, cognitive modulation

## Abstract

**Objective:** This study aimed to explore the acute neurophysiological effects of a single oral dose of Astragaloside IV (AS-IV) on EEG-measured brain oscillations and cognitive-relevant spectral markers in healthy young adults. **Methods:** Twenty healthy adults (8 females, 12 males; mean age: 23.4±2.1) underwent eyes-closed resting-state EEG recordings before and approximately 90 min after oral intake of 150 mg AS-IV. EEG data were collected using a 21-channel 10–20 system and cleaned via Artifact Subspace Reconstruction and Independent Component Analysis. Data quality was confirmed using a signal-to-noise ratio and 1/f spectral slope. Absolute and relative power values, band ratios, and frontal alpha asymmetry were computed. Statistical comparisons were made using paired *t*-tests or Wilcoxon signed-rank tests. **Results:** Absolute power decreased in delta, theta, beta, and gamma bands (*p* < 0.05) but remained stable for alpha. Relative alpha power increased significantly (*p* = 0.002), with rises in relative beta, theta, and delta and a drop in relative gamma (*p* = 0.003). Alpha/beta and theta/beta ratios increased, while delta/alpha decreased. Frontal alpha asymmetry was unchanged. Sex differences were examined in all measures that showed significant changes; however, no sex-dependent effects were found. **Conclusions:** A single AS-IV dose may acutely modulate brain oscillations, supporting its potential neuroactive properties. Larger placebo-controlled trials, including concurrent psychometric assessments, are needed to verify and contextualize these findings. A single AS-IV dose may acutely modulate brain oscillations, supporting its potential neuroactive properties.

## 1. Introduction

Medicinal plants have long been used worldwide as both traditional and strategic tools in the management of various health conditions [1]. The identification of approximately 12,000 medicinal plants [2] has increased scientific interest in the potential health effects of these plants. Among them, Astragalus membranaceus (AM) is a well-established herb that has been used for over 2000 years in traditional Chinese medicine and is extensively described in the Chinese Pharmacopoeia for its diverse pharmacological properties, notably its antioxidant, anti-inflammatory, and immunoregulatory effects [3,4]. The primary bioactive constituents of AM include polysaccharides, saponins, and flavonoids, whose content may vary depending on geographic origin [5].

Astragaloside IV (AS-IV) has shown neuroprotective effects in Alzheimer’s and Parkinson’s disease models, particularly against learning and memory impairments induced by glucocorticoid-mediated stress [6,7]. These impairments are often linked to “cold” executive functions such as working memory, attention regulation, response inhibition, and cognitive flexibility—domains that are among the earliest affected in both Parkinson’s disease (PD) and Alzheimer’s disease (AD) [8,9,10]. Protecting or modulating these specific cognitive domains through safe and bioavailable compounds like AS-IV offers a promising avenue for early intervention strategies. AS-IV has been reported to have a low toxicity profile and does not adversely affect liver or kidney functions when administered orally [11]. Moreover, experimental ischemic models have demonstrated that AS-IV can cross the blood–brain barrier [12]. The mild organoleptic properties of AM extract make it suitable for incorporation into functional foods [13]. These features position AS-IV as a promising dietary supplement candidate for cognitive health, particularly in neurodegenerative disorders.

In neuroscience research, various neurophysiological measurement techniques are employed to assess the acute effects of herbal bioactive compounds on brain function. Among these techniques, electroencephalography (EEG) is a cost-effective, accessible, and widely used method for evaluating brain wave activity. EEG analyzes frequency-based brain activity through delta (1–4 Hz, linked to attention and top-down control), theta (4–8 Hz), alpha (8–12 Hz, relaxation and alertness), beta (12–30 Hz, task-related engagement), and gamma (30–45 Hz, integration of sensory and cognitive functions) bands, which are, respectively, associated with deep sleep, light sleep and creativity, relaxation and learning, focus and problem solving, and cognitive integration [14,15]. Recent findings suggest that changes in delta and theta power may reflect cognitive and perceptual mechanisms in neurodevelopmental contexts [16]. Several studies have investigated the acute effects of natural compounds on EEG activity. The use of rosemary has been suggested to have positive effects on cognitive functions [17], while the consumption of green and black tea has been examined for its impact on alertness [18]. Similarly, other studies have observed the sedative effects of inhaled lavender oil [19] and tangerine oil [20]. However, the neurophysiological impact of AS-IV on brain oscillations has not yet been thoroughly investigated.

The current study aims to contribute to the existing literature by investigating the acute effects of orally administered AS-IV on resting-state EEG spectral power in healthy individuals. Given AS-IV’s reported cognitive and neuroprotective properties, we hypothesize that AS-IV may modulate EEG patterns in a manner consistent with enhanced attention, working memory, or cognitive alertness. The findings of this pilot study aim to provide preliminary evidence for the neuromodulatory properties of AS-IV and lay the groundwork for future randomized, placebo-controlled clinical trials integrating cognitive and psychometric assessments.

## 2. Materials and Methods

### 2.1. Participants

To determine the minimum required sample size, an a priori power analysis was conducted using G*Power 3.1.9.7. The analysis indicated that 12 participants would be sufficient to detect a large effect size (Cohen’s d=0.8) with 80% power in a two-tailed paired-samples *t*-test at an alpha level of 0.05. Therefore, the inclusion of 20 participants in this study provided a wider safety margin and increased sensitivity for detecting within-subject changes in this exploratory EEG investigation. Although some outcome variables violated the assumption of normality and were analyzed using the non-parametric Wilcoxon signed-rank test, the power analysis based on the paired *t*-test remains appropriate given the within-subject design and the large expected effect size.

A total of twenty healthy volunteers (eight females and twelve males; 40% female, 60% male), aged between 19 and 27 years (mean age: 23.4 ± 2.1), participated in this study. Participant characteristics are summarized in Table 1. All participants were undergraduate students from Istanbul Nişantaşı University, ensuring demographic homogeneity in terms of age, education, and lifestyle. Therefore, although no statistical analysis was performed based on clinical variables, such as cultural background or education level, the characteristics of the sample inherently minimized variability in these dimensions. The research protocol was approved by the Ethics Committee of Istanbul Nişantaşı University (Protocol Code: 2020/23; Approval Date: 8 December 2020), and written informed consent was obtained from all participants. Individuals with any physiological or psychiatric conditions that might affect the study outcomes were excluded. Additionally, it was verified that none of the participants used any medication or substances that could influence metabolic parameters, and all participants were non-smokers.

This study aimed to evaluate the acute cognitive effects of AS-IV in individuals with no pre-existing medical conditions. AS-IV (purity ≥ 98%, GMP-certified API) was obtained from Chengdu Biopurify Phytochemicals Ltd. (Chengdu, China) and was administered to each participant as a single oral dose of AS-IV dissolved in water.

In this study, an experimental protocol of approximately 90 min was conducted for each volunteer. Initially, participants’ spontaneous brain activity was recorded using EEG. Following the baseline recording, each participant received a single oral dose of AS-IV, and an absorption and acute effect observation period of 90 min—consistent with the compound’s half-life [21]—was allowed. After this period, a second EEG recording was performed, and the acquired data were comprehensively analyzed.

### 2.2. Dose Determination

The dose selection for AS-IV in this study was based primarily on previous animal studies. In rodent models, AS-IV administered at 10–20 mg/kg/day has been reported to exert significant neuroprotective effects. For instance, in models of chronic cerebral hypoperfusion, a dose of 20 mg/kg/day improved learning and memory functions, while in brain injury models, intraperitoneal administration of 20 mg/kg/day reduced oxidative stress and inflammation [22]. Furthermore, toxicity studies have demonstrated that LS-102, a derivative of AS-IV, caused no notable adverse effects even at very high doses up to 5000 mg/kg in mice [23]. Although a comparable high-dose toxicity study specifically for AS-IV is lacking, the fact that LS-102 retains the pharmacological core structure of AS-IV suggests that the two compounds likely share similar pharmacodynamic targets and tolerability profiles. Therefore, the high-dose tolerance observed with LS-102 indicates that AS-IV may also possess a wide safety margin.

When translating doses from animal models to humans, an allometric conversion based on body surface area (BSA) was employed instead of a simple weight-based scaling. This method aligns with FDA and ICH guidelines and is widely recognized, as outlined by Reagan-Shaw et al. (2008) [24].

Considering all these data, although no pre-established safe dose exists for humans, a single oral dose of 150 mg AS-IV was selected to ensure observable pharmacodynamic effects while maintaining a low risk profile. This decision is consistent with dose-escalation protocols reported in early-phase human studies of comparable herbal saponins [25,26].

### 2.3. Experimental Process

The experimental protocol lasted approximately 90 min per participant. Each session began with a 15 min baseline EEG recording of spontaneous brain activity. After this baseline measurement, participants orally ingested AS-IV. A subsequent 90 min waiting period was applied to allow for the absorption of AS-IV and the manifestation of its acute effects, based on the known half-life of the compound [21]. Following this period, a second EEG recording was conducted under the same conditions. Although no placebo group was included in this initial pilot study, the within-subject design was considered appropriate for detecting short-term spectral changes attributable to AS-IV intake in a controlled EEG environment.

### 2.4. EEG Recording and Preprocessing

EEG signals were recorded using the international 10–20 electrode placement system with 21 channels, employing a Micromed SD Plus Flexi system (Micromed SpA, Mogliano Veneto, Treviso, Italy). The right earlobe (A2) electrode served as the reference. All recordings were obtained in a resting-state condition while participants sat in a quiet, dimly lit, and temperature-controlled room. Subjects were instructed to remain relaxed with their eyes closed throughout the session.

EEG data were sampled at 256 Hz and filtered using a 1–45 Hz finite impulse response (FIR) bandpass filter. Noise rejection was implemented using a combination of Artifact Subspace Reconstruction (ASR) and Independent Component Analysis (ICA), both integrated within the EEGLAB toolbox [27,28]. To improve ICA performance and minimize the effects of volume conduction in spectral analyses, a surface Laplacian referencing scheme was applied, a method shown to enhance spatial resolution by isolating local cortical activity [29].

Following ICA decomposition, independent components were automatically classified using the ICLabel algorithm [30] and the Multiple Artifact Rejection Algorithm (MARA) [31]. Components with an ICLabel “brain” probability < 0.75 or a MARA probability > 0.5 were considered artifacts and removed.

To evaluate the quality of the cleaned EEG data, the signal-to-noise ratio (SNR) was calculated after artifact rejection. Although no universally accepted threshold exists for EEG SNR, values above 10 dB are commonly considered indicative of high-quality recordings, while values between 5 and 10 dB are interpreted as moderately clean. This classification is supported by prior studies employing SNR as a criterion for evaluating EEG data quality [32]. The power spectral density (PSD) standard error of the mean was also examined to assess the consistency and residual noise across recordings. Furthermore, the 1/f spectral slope was estimated by fitting a linear regression line to the log–log-transformed power spectral density (PSD) curve, calculated using the Welch method (2 s window, 50% overlap). The averaged pre- and post-intervention PSDs, along with the expected 1/f spectral profile, were plotted on a log–log scale to allow for a visual inspection of noise suppression and signal integrity.

### 2.5. Spectral Analysis

The preprocessed EEG data were subjected to PSD analysis using MATLAB R2021a (version 9.10)’s built-in pwelch function [33,34]. PSD estimates were computed for each participant, channel, and condition using a 2-s Hamming window with 50% overlap, providing an optimal trade-off between frequency resolution and estimation stability. The resulting PSD values were segmented into canonical frequency bands: delta (1–4 Hz), theta (4–8 Hz), alpha (8–12 Hz), beta (12–30 Hz), and gamma (30–45 Hz). Absolute band powers were calculated as the area under the curve within each band, and relative powers were computed by dividing each band power by the total power across 1–45 Hz. Additionally, spectral ratios such as alpha/beta, delta/alpha, theta/beta, and beta/gamma were computed by dividing the absolute power values of the corresponding frequency bands. Frontal alpha asymmetry (FAA) was calculated as the natural logarithmic difference in absolute alpha power between F4 and F3 electrodes, defined as $\ln\left(P_{\alpha ,F4}\right)-\ln\left(P_{\alpha ,F3}\right)$.

Channels exhibiting excessive noise were excluded from the analysis. However, participants with globally poor EEG quality were not removed entirely to preserve the dataset size. Instead, targeted preprocessing procedures were applied to mitigate noise effects. Despite these efforts, moderate residual noise led to the occurrence of extreme values in some recordings, which disproportionately affected descriptive statistics such as the mean, standard deviation (SD), and variance. To address this issue, a winsorization procedure was employed [35], limiting the influence of outliers and providing a more robust estimation of central tendency and variability.

### 2.6. Statistical Analyses

As most grand-averaged spectral variables violated the assumption of normality, comparisons between pre- and post-intervention conditions were primarily conducted using the Wilcoxon signed-rank test. This non-parametric approach was applied to absolute power, relative power, and spectral power ratios (e.g., alpha/beta, delta/alpha, alpha/theta, theta/beta, beta/gamma).

To ensure appropriate statistical testing in the presence of moderate noise and limited sample size, we adopted a hybrid approach using both parametric and non-parametric tests based on distributional assumptions. For each frequency band and channel pair, normality was tested using the Shapiro–Wilk test. If the distribution of pre–post spectral differences did not significantly deviate from normality (*p* > 0.05), paired-samples *t*-tests were used. Otherwise, the Wilcoxon signed-rank test was applied (Table S1). This hybrid approach is used in the literature [36] and allowed for a more robust analysis under varying data characteristics [37], which is particularly relevant in EEG research due to inter-individual variability and potential deviations from ideal data distributions [35,38]. For transparency, a summary table listing the normality test results, test type, and sample size for each comparison is included in the Supplementary Materials. To account for the risk of false positives due to multiple comparisons (21 channels × 6 frequency bands), *p*-values were corrected using the Benjamini–Hochberg False Discovery Rate (FDR) procedure (*q* < 0.05). Effect sizes were reported using Cohen’s d and r value. Furthermore, exploratory comparisons between groups (male versus female) were conducted post hoc to examine potential sex-related differences in the current results. A similar hybrid statistical approach was used: normality was assessed using the Shapiro–Wilk test, and either an independent-samples *t*-test or a Mann–Whitney U test was applied depending on the outcome. To control for multiple comparisons, *p*-values were adjusted using the Benjamini–Hochberg False Discovery Rate (FDR) procedure (*q* < 0.05). The full results are provided in the Supplementary Materials and summarized in the Section 3. All statistical analyses were performed in MATLAB.

## 3. Results

After automated data cleaning based on ASR and ICA, the SNR was calculated to evaluate residual noise and estimate the loss of potentially meaningful information. The average SNR was found to be 12.75 ± 11.00, with a maximum value of 66.25 and a minimum of −0.12. SNR values above 10 dB are generally considered to reflect sufficiently cleaned data, while values below this threshold indicate moderate noise contamination [32]. To mitigate the impact of outliers, the median absolute deviation (MAD) was also computed, yielding an average of 10.21 ± 5.65. These findings indicate that the overall noise level was moderate, and this was taken into account during statistical analyses.

**Figure 1:**
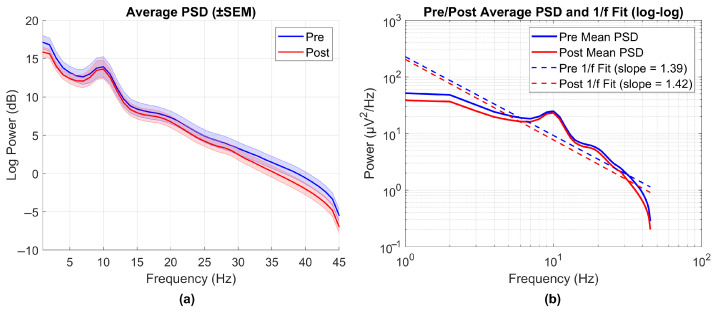
PSD graphs and corresponding 1/f power distribution fits are presented to assess noise characteristics in the EEG signal. (**a**) Mean power spectral density (PSD) plots of EEG signals before (pre) and after (post) the intervention, with shaded areas representing ± standard error of the mean (SEM). (**b**) The same PSD data are plotted in log–log scale, with linear fits illustrating the alignment with the expected 1/f power distribution. This panel is intended to visualize the preservation of the characteristic 1/f structure of neural signals.

When the average PSD values shown in Figure 1a are examined, it is seen that the error rate is at an acceptable level and no sudden changes are observed. This is consistent with the SNR examination and shows that the data can be evaluated in terms of average power. It is also seen that the average PSD decreases except for the alpha band in the process following use. In addition to this assessment, the 1/f power spectrum curve is presented in Figure 1b. When the distribution of EEG signal power against frequency is examined on a log–log scale, it is observed that the classical 1/f decay trend is preserved, with no major deviations from this pattern. Although the slope of the curve varies across individuals, the average slope values (pre 1/f fit slope = 1.39, post 1/f fit slope = 1.42) are consistent with typical 1/f characteristics and do not indicate the presence of artificial noise or systematic error. This supports the physiological validity of the overall spectral structure and confirms that the signal is suitable for further analysis.

**Figure 2:**
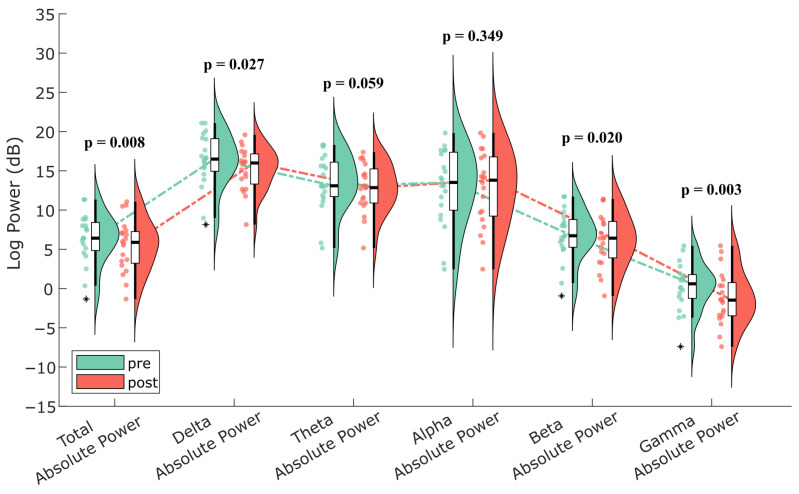
Violin plot illustrating the comparison of absolute power values across all frequency bands before (pre) and after (post) the intervention. The results of the Wilcoxon signed-rank test are presented in the figure. A significant decrease was observed in total absolute power (*p* = 0.008), while fewer changes were observed in the frequency bands. The thick central line represents the median, the width reflects the distribution density, and the dots indicate individual participant data, and asterisks (*) indicate outliers. Dashed lines link within-group medians across frequency bands to visualize spectral trends separately for the pre and post conditions.

In spectral power analyses, Wilcoxon signed-rank tests applied to pre- and post-intervention comparisons revealed a significant reduction in total absolute power (*p* = 0.008) (Figure 2). No significant change was observed in absolute alpha power, while reductions were detected in other bands: delta (*p* = 0.027), theta (*p* = 0.059), beta (*p* = 0.020), and gamma (*p* = 0.003). These changes are visualized in the violin plot shown in Figure 2, which illustrates the distribution and medians of spectral power across frequency bands. To aid interpretation, lines connecting the medians within each condition are included to highlight the variation across frequency bands.

**Figure 3:**
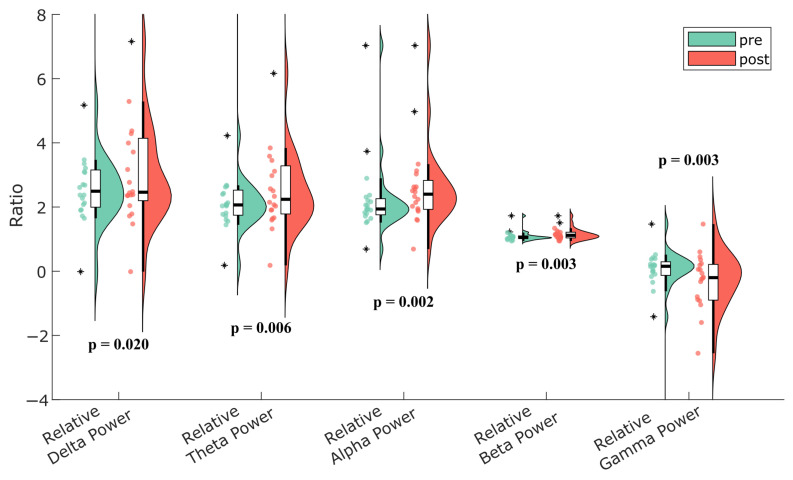
Violin plot illustrating the comparison of relative power values across all frequency bands before (pre) and after (post) the intervention. The Wilcoxon signed-rank test results are presented in the figure. A significant increase was observed in the alpha band (*p* = 0.002), with milder increases in beta (*p* = 0.003), theta (*p* = 0.006), and delta (*p* = 0.020) bands, while gamma power showed a significant decrease (*p* = 0.003). The thick central line represents the median, the width reflects the distribution density, and the dots indicate individual participant data, and asterisks (*) indicate outliers.

To account for possible baseline noise effects, relative power values were additionally examined. The most prominent increase was observed in the alpha band (*p* = 0.002), followed by increases in relative beta (*p* = 0.003), theta (*p* = 0.006), and delta (*p* = 0.020) power. In contrast, relative gamma power significantly decreased (*p* = 0.003). These relative power changes are illustrated in Figure 3.

**Figure 4:**
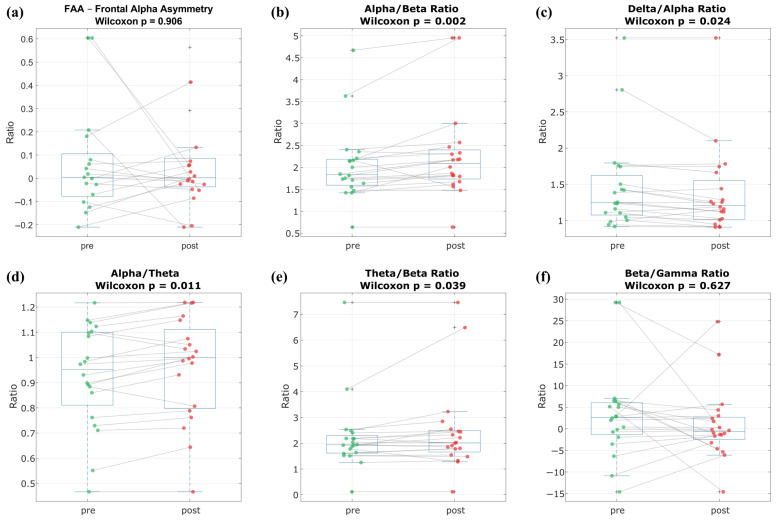
Boxplots showing the comparison of band power ratios before (pre) and after (post) the intervention. According to the Wilcoxon signed-rank test results, a significant increase was observed in the alpha/beta (*p* = 0.002) and theta/beta (*p* = 0.039) ratios, while a significant decrease was observed in the delta/alpha ratio (*p* = 0.024). No significant change was observed in the FAA and beta/gamma ratio (*p* = 0.627). Boxes represent the median, interquartile range, and potential outliers. Dots indicate individual participant data, and ”+” indicates outliers. Light gray lines depict subject-level changes across conditions, highlighting inter-individual variability in response to the intervention.

Among the frequency band ratios that are widely discussed in the literature due to their associations with various physiological effects, no significant change was observed in FAA (Figure 4a). However, significant increases were found in the alpha/beta and alpha/theta ratios (*p* = 0.002 and *p* = 0.011, respectively), as shown in Figure 4b and Figure 4d. A significant decrease was observed in the delta/alpha ratio (*p* = 0.024) (Figure 4c), while the theta/beta ratio also showed a significant increase (*p* = 0.039) (Figure 4e). No significant change was found in the beta/gamma ratio (*p* = 0.627) (Figure 4f).

To enhance interpretability and support potential meta-analyses, descriptive statistics were computed for each EEG metric, including the mean and standard error of the mean (SE) before and after AS-IV administration. In addition to *p*-values obtained from Wilcoxon signed-rank tests, standardized z-values and effect sizes r were calculated to quantify the magnitude and direction of the observed changes. These results are summarized in Table 2.

The reported values are based on winsorized data, which were also used for all statistical testing to reduce the influence of outliers and improve robustness. This approach was preferred for power-related statistics, as excluding outlier values entirely could reduce sample representativeness, whereas winsorization preserves the data structure while limiting the undue influence of extreme values on the mean. Importantly, this procedure allowed the inclusion of all participants in the analysis, ensuring that no subject was excluded due to data-driven thresholds. A complete summary of all tested metrics is provided in Table 2.

Channel–frequency pair analyses that showed significant changes are presented in Table 3. Considering the moderate noise present in the recordings, localized channel–frequency findings were evaluated with increased scrutiny to ensure the robustness of the findings. Paired *t*-tests were used for pairs that met the assumption of normality, while Wilcoxon signed-rank tests were applied to those that did not. To monitor the potential impact of channel removal during artifact correction, the number of participants included in each comparison is reported in Table 3. The Fp1 electrode was excluded from the analysis due to insufficient data.

Additionally, a total of 126 statistical comparisons were conducted, and to reduce the risk of Type I error due to multiple testing, FDR correction was applied using the Benjamini–Hochberg procedure. However, no statistically significant results were found under the threshold of *q* < 0.05. Although localized analyses have inherent limitations due to the nature of the dataset, the findings are reported here as they may offer valuable insights for future research.

To explore potential sex-related effects, all outcome measures (absolute and relative power, band ratios, FAA, and band–channel pairs) were compared between male and female participants using independent-samples *t*-tests or Mann–Whitney U tests, depending on normality. No statistically significant differences were observed across any of the tested measures, even prior to applying the FDR correction. The full results are provided in the Supplementary Materials (Table S2).

## 4. Discussion

In this study, the acute effects of AS-IV on resting-state EEG activity in healthy individuals were investigated and evaluated based on spectral power measures. The findings showed that, in terms of absolute power, there was a significant decrease in all frequency bands except for the alpha band; delta, theta, beta, and gamma bands exhibited statistically significant reductions. In the relative power analysis, the most prominent change was a significant increase in the alpha band, followed by increases in relative beta, theta, and delta powers. In contrast, relative gamma power showed a significant decrease. Detailed analyses at the channel-frequency level revealed statistically significant changes across delta, theta, beta, and gamma bands. These effects were observed in various EEG channels, suggesting that AS-IV may modulate cortical oscillations; however, region-specific patterns were not the primary focus and require further study.

The findings of this study indicate that while absolute band powers generally decreased, relative power values—based on the ratio of each band power to total power—showed statistically significant changes. This pattern may be attributable to variations in baseline noise levels and differences in SNR during measurement. To address this, SNR values and the fit of the 1/f power distribution curve were examined, confirming that the signal contained an acceptable level of moderate noise. The calculated band-to-total power ratios were intended to compensate for this effect. Therefore, in interpreting the results, it was considered more appropriate to focus on relative power values rather than absolute values, as they better reflect the distribution of spectral activity within the total power, especially given the relatively wide range of baseline noise observed in this dataset. Nonetheless, the pattern observed in the absolute power values—with decreases in delta, theta, beta, and gamma bands, but relatively stable alpha power—may also indicate meaningful physiological insights. Following AS-IV intake, the findings may indicate a downregulation of cortical arousal. This interpretation aligns with previous research showing that increased beta and gamma activity is associated with heightened arousal and stress responses [39,40], whereas stable or elevated alpha activity reflects a relaxed yet alert mental state [41,42]. The absence of a significant change in alpha power may suggest that AS-IV helps preserve wakeful relaxation while suppressing overactivation in higher frequency bands.

Alpha band oscillations reflect one of the most fundamental cognitive processes and also play a key role in the integration of brain activities across different frequencies [43]. An increase in alpha waves has been associated with heightened attention and alertness. Refs. [18,44,45] reported an increase in alpha brain waves following the consumption of certain dietary components found in tea. Similarly, Lagopoulos et al. (2009) observed significant increases in alpha power during studies on non-directive meditation and linked this to a state of relaxation [44]. Some studies have also reported that high alpha power increases when working memory load decreases and anxiety is reduced [43,46]. The observed increase in relative alpha may thus indicate a cortical arousal state characterized by relaxed wakefulness and internalized attention, which can support cognitive readiness.

Although no change was observed in FAA, the alpha/beta ratio showed a significant increase (*p* = 0.002), the delta/alpha ratio demonstrated a significant decrease (*p* = 0.024), and the theta/beta ratio increased significantly (*p* = 0.039); no significant change was found in the beta/gamma ratio (*p* = 0.627).

The alpha/beta ratio can be used to analyze shifts in an individual’s cognitive state [47]. This ratio is strongly associated with stress physiology and anxiety states: a lower alpha/beta ratio is generally seen in conditions of excessive mental workload, high cognitive arousal, and stress. An increase in the ratio indicates a reduction in mental stress and the activation of cortical inhibitory mechanisms [48]. In the clinical EEG literature, an increase in the alpha/beta ratio is generally linked to anxiolytic effects [49,50].

In aging and pathological conditions, resting-state delta power is typically increased, indicating cortical slowing [51]. In our study, the significant decrease in the delta/alpha ratio (*p* = 0.024) observed after AS-IV administration may suggest that AS-IV intake has a positive effect on mental clarity and attention levels.

The alpha/theta ratio has been highlighted in research as a valuable tool, with its diagnostic accuracy shown to be higher than that of visual EEG inspection [52]. A low alpha/theta ratio is commonly observed in dementia and cognitive impairments [53]. In contrast, in our study, the observed increase in the alpha/theta ratio following AS-IV intake aligns with an optimal cognitive functioning and a healthier cortical activity pattern. Therefore, it can be interpreted that the acute use of AS-IV may support cognitive stability and neurophysiological health in healthy young adults.

Salma et al. (2017) demonstrated that a higher theta/beta ratio is associated with increased stress levels, suggesting its potential as an indicator of stress during cognitive tasks with varying levels of difficulty [54]. Additionally, the theta/beta ratio was initially introduced as an EEG marker for diagnosing attention deficit/hyperactivity disorder (ADHD) in relation to arousal deficits [55]. This ratio has also been linked to various aspects of executive cognitive performance in healthy individuals [56]. Experimental results from another study showed that both the alpha/beta and theta/beta ratios decreased after exposure to a stress stimulus, indicating a more stressed state [57].

There are two types of frontal EEG alpha asymmetry, resting-state frontal EEG alpha asymmetry and frontal EEG alpha asymmetry, which are measured during task conditions involving emotional challenges. Resting-state frontal EEG alpha asymmetry is associated with various trait-like individual differences and is therefore also referred to as trait frontal EEG alpha asymmetry [58]. In our study, no statistically significant change was observed in FAA at rest. FAA reflects stable individual differences and is relatively resistant to short-term experimental manipulations [59]. Therefore, acute pharmacological or herbal interventions may not immediately alter FAA, although general alpha power and band ratios may change.

Among the relative power measures, the alpha band showed the most significant increase (*p* = 0.002). This was followed by increases in relative beta (*p* = 0.003), theta (*p* = 0.006), and delta (*p* = 0.020) power, while relative gamma decreased (*p* = 0.003). Absolute power reflects the raw energy level within each frequency band, whereas relative power indicates the proportion of each band’s power relative to total brain activity. The observed pattern, characterized by increased alpha/beta ratio and gamma suppression, suggests that AS-IV intake may reduce cortical arousal and promote a state of mental relaxation and internalized attention. Such EEG changes are consistent with neurophysiological profiles described in previous studies of stress-buffering and anxiolytic interventions, including mindfulness practices, neurofeedback, and pharmacological treatments [60,61,62,63,64].

In our study, gamma wave frequency exhibited a notable reduction, particularly in the central regions. This decrease in gamma activity suggests a reduction in mental stress, a lightening of cognitive load, and an enhancement of relaxation levels. The observed central concentration supports the hypothesis that changes in gamma waves indicate a more relaxed and focused mental state [14]. Furthermore, this finding implies that AS-IV may potentially exert anxiolytic or mild neuro-sedative effects. This interpretation aligns with previous preclinical studies demonstrating that AS-IV shows neuroprotective and stress-reducing effects in glucocorticoid-induced stress models [7,25,65].

The modulation observed in gamma activity suggests that AS-IV intake may regulate levels of attention and arousal by optimizing synchronization within cortical information processing networks. This finding is consistent with Fries’ “communication through coherence” (CTC) hypothesis [66,67], which proposes that gamma synchronization facilitates information transfer, whereas excessive gamma activity is known to be associated with stress and heightened mental load. Therefore, the modulation of gamma by AS-IV could be related to balanced arousal levels and a potential anxiolytic effect.

The findings of this study support the hypothesis that AS-IV intake may exert beneficial effects on brain function in individuals without prior neurological conditions. Based on the current data, AS-IV holds potential as an adjunct in the management of mental health conditions, such as attention deficit disorders, and may also play a supportive role in cognitive decline, including Alzheimer’s disease. However, this study should be regarded as an initial step toward understanding the effects of AS-IV on brain function. The results provide an important foundation for future research aiming to further elucidate the therapeutic potential of AS-IV and its contributions to brain health and overall well-being. Therefore, these findings serve as an essential starting point for more comprehensive investigations designed to clarify the neurophysiological mechanisms and clinical benefits of AS-IV in larger and controlled human studies.

Despite promising findings, this study has several limitations that should be acknowledged and addressed in future research. First, the absence of a placebo-controlled, double-blind design restricts the ability to draw strong causal inferences and introduces the possibility of expectancy or nonspecific effects. However, open-label, within-subject exploratory designs are commonly used in early-phase phytopharmacological research. Similar methodological approaches have been employed in studies on Bacopa monnieri, which, despite lacking placebo groups, yielded valuable biological insights and guided future clinical investigations [68,69]. Second, although post hoc power analysis suggested sufficient sensitivity to detect large effects (Cohen’s d), the relatively small sample size (n = 20) still limits the generalizability and statistical robustness of the findings. Future studies should be designed to detect smaller effect sizes with adequate power. Third, the absence of concurrent behavioral assessments or cognitive task performance data limits the interpretation of the functional significance of EEG changes. Moreover, the lack of validated psychometric measures of stress or anxiety prevents any affective or emotional interpretations of the neurophysiological alterations observed. Future research should address these limitations by incorporating placebo-controlled, double-blind designs with larger and more diverse samples, along with cognitive or emotional assessments to relate EEG findings to behavioral outcomes.

## 5. Conclusions

Our findings indicate that AS-IV has the potential to exert rapid neurophysiological effects by modulating cortical oscillations measurable via EEG. This supports the prospect of AS-IV being utilized as a functional food component or herbal supplement to promote brain health. The preliminary results suggest that AS-IV intake may have beneficial effects on brain function in healthy individuals and could provide supportive benefits in conditions such as attention deficit disorders or cognitive decline. To better understand the relationship between AS-IV’s neurophysiological effects and associated cognitive, emotional, and behavioral outcomes, larger-scale, placebo-controlled, double-blind studies are necessary. Overall, these results provide a valuable basis for future research aimed at elucidating the therapeutic potential of AS-IV and its contributions to brain health and well-being.

## Figures and Tables

**Table 1:** Description of the study population.

Characteristics	Informations
Participant Number	20
Laterality	100% right-handed
Gender	40% female
Age (years)	23.4 ± 2.1
Weight (kg)	63.1 ± 10.7
BMI	21.52 ± 1.87
Literacy rate	100%

Values are represented using mean ± standard deviation.

**Table 2:** Descriptive statistics and test results for EEG spectral metrics before and after AS-IV administration.

EEG Metric	Pre (Mean ± SE)	Post (Mean ± SE)	Test	*p*-Value	*z*-Value	*r*-Value
Total Power	6.341 ± 0.721 *	5.478 ± 0.745 *	wilcoxon	0.008	2.66	0.59
Delta Power	16.298 ± 0.781 *	15.226 ± 0.615 *	wilcoxon	0.027	2.21	0.49
Theta Power	13.499 ± 0.817 *	12.797 ± 0.700 *	wilcoxon	0.059	1.89	0.42
Alpha Power	12.948 ± 1.074 *	12.752 ± 1.072 *	wilcoxon	0.349	0.94	0.21
Beta power	6.765 ± 0.739 *	6.169 ± 0.767 *	wilcoxon	0.020	2.33	0.52
Gamma Power	0.225 ± 0.685 *	−1.194 ± 0.762 *	wilcoxon	0.003	2.98	0.67
Relative Delta	3.204 ± 0.726	3.624 ± 0.752	wilcoxon	0.020	−2.33	−0.52
Relative Theta	2.542 ± 0.492	2.916 ± 0.520	wilcoxon	0.006	−2.72	−0.61
Relative Alpha	2.264 ± 0.283	2.633 ± 0.300	wilcoxon	0.002	−3.11	−0.7
Relative Beta	1.092 ± 0.037	1.157 ± 0.042	wilcoxon	0.003	−2.98	−0.67
Relative Gamma	−0.246 ± 0.353	−0.598 ± 0.368	wilcoxon	0.003	2.98	0.67
FAA	0.065 ± 0.056	0.005 ± 0.033	wilcoxon	0.906	0.12	0.03
Alpha/Beta	2.036 ± 0.189	2.268 ± 0.232	wilcoxon	0.002	−3.06	−0.68
Delta/Alpha	1.467 ± 0.145	1.379 ± 0.135	wilcoxon	0.024	2.25	0.50
Alpha/Theta	0.927 ± 0.045	0.961 ± 0.046	wilcoxon	0.011	−2.54	−0.57
Theta/Beta	2.232 ± 0.320	2.457 ± 0.377	wilcoxon	0.039	−2.07	−0.46
Beta/Gamma	3.334 ± 2.362	0.928 ± 1.836	wilcoxon	0.627	0.49	0.11

Note. * indicates power values expressed in decibels (dB), computed as $10\cdot \log_{10}\left(\mu V^{2}\right)$. Other values are unitless.

**Table 3:** Statistical results for EEG power comparisons across frequency band–channel pairs. Paired *t*-tests were applied to normally distributed data, and Wilcoxon signed-rank tests were used otherwise. The table presents *p*-values, the number of participants (*N*), and the corresponding test statistic (*t* or *z*), depending on whether a parametric or non-parametric test was applied. Effect sizes are reported as Cohen’s *d* for *t*-tests and as *r* for non-parametric tests. Means are expressed as mean ± standard error (SE). Only combinations with statistically significant differences (p<0.05) are included. All power values are expressed in decibels (dB), computed as $10\log_{10}\left(\mu V^{2}\right)$.

Band	Ch	Pre (M ± SE)	Post (M ± SE)	Test	*p*	*t*/*z*	*d*/*r*	N	*q*
All	Cz	5.236 ± 0.519	4.405 ± 0.701	wilcoxon	0.015	2.43	0.54	20	0.175
All	F7	5.874 ± 1.268	4.468 ± 0.965	wilcoxon	0.040	2.05	0.47	19	0.241
All	Fp2	5.417 ± 0.971	3.683 ± 1.272	paired *t*-test	0.048	2.13	−0.50	18	0.241
All	Fz	6.947 ± 0.716	5.963 ± 0.636	paired *t*-test	0.032	2.31	−0.52	20	0.241
All	T4	5.734 ± 1.014	3.401 ± 0.993	paired *t*-test	0.004	3.47	−0.90	15	0.142
All	T6	6.193 ± 0.956	4.649 ± 1.122	paired *t*-test	0.012	2.83	−0.69	17	0.153
Alpha	P4	16.183 ± 1.489	15.230 ± 1.657	paired *t*-test	0.048	2.14	−0.52	17	0.241
Beta	T4	5.953 ± 1.004	3.802 ± 0.977	paired *t*-test	0.006	3.27	−0.84	15	0.142
Beta	T6	6.547 ± 0.960	5.195 ± 1.145	paired *t*-test	0.020	2.57	−0.62	17	0.184
Delta	Cz	16.270 ± 0.610	14.693 ± 0.690	wilcoxon	0.008	2.65	0.59	20	0.145
Delta	Fp2	16.550 ± 1.186	13.985 ± 1.354	paired *t*-test	0.039	2.24	−0.53	18	0.241
Delta	FpZ	17.375 ± 1.429	14.126 ± 1.695	paired *t*-test	0.045	2.23	−0.62	13	0.241
Delta	Fz	18.837 ± 0.766	17.342 ± 0.580	paired *t*-test	0.018	2.58	−0.58	20	0.179
Delta	OZ	17.373 ± 0.629	15.509 ± 0.696	paired *t*-test	0.005	3.27	−0.77	18	0.142
Delta	Pz	17.647 ± 0.628	15.505 ± 1.029	wilcoxon	0.017	2.39	0.53	20	0.177
Gamma	Cz	−2.165 ± 0.478	−3.283 ± 0.739	wilcoxon	0.004	2.87	0.64	20	0.142
Gamma	F7	1.021 ± 1.393	−1.348 ± 1.130	paired *t*-test	0.043	2.17	−0.50	19	0.241
Gamma	Fz	−0.364 ± 0.604	−1.660 ± 0.581	paired *t*-test	0.009	2.90	-0.65	20	0.145
Gamma	Pz	−1.638 ± 0.481	−2.961 ± 0.956	wilcoxon	0.044	2.02	0.45	20	0.241
Gamma	T4	0.879 ± 1.217	−2.522 ± 1.162	paired *t*-test	0.004	3.40	−0.88	15	0.142
Gamma	T5	0.624 ± 1.273	−0.508 ± 1.259	wilcoxon	0.044	2.01	0.49	17	0.241
Gamma	T6	0.033 ± 1.071	−1.939 ± 1.178	paired *t*-test	0.011	2.88	−0.70	17	0.151
Theta	Cz	14.475 ± 0.708	13.267 ± 0.789	wilcoxon	0.009	2.61	0.58	20	0.145
Theta	Fp2	13.036 ± 1.066	11.138 ± 1.198	paired *t*-test	0.033	2.32	−0.55	18	0.241

## Data Availability

The data presented in this study are available upon reasonable request from the corresponding author.

## Supplementary Materials

The following supporting information can be downloaded at: https://www.mdpi.com/article/10.3390/nu17152425/s1, Table S1: Normality test results and corresponding statistical test selection for each band–channel pair; Table S2: Statistical analyses evaluating sex-related effects in EEG features with significant pre–post differences.

## Abbreviations

The following abbreviations are used in this manuscript:

AS-IV	Astragaloside IV
AM	Astragalus membranaceus
EEG	Electroencephalography
SNR	Signal-to-noise ratio
ASR	Artifact Subspace Reconstruction
ICA	Independent Component Analysis
FAA	Frontal alpha asymmetry
FDR	False Discovery Rate
BSA	Body surface area
FDA	Food and Drug Administration
ICH	International Council for Harmonization
FIR	Finite impulse response
MARA	Multiple Artifact Rejection Algorithm
SD	Standard deviation
MAD	Median absolute deviation
ADHD	Attention deficit/hyperactivity disorder
CTC	Communication through coherence
