# Supplementary material for: A Pilot EEG Study on the Acute Neurophysiological Effects of Single-Dose Astragaloside IV in Healthy Young Adults
Source: Nutrients. 2025 Jul 24;17(15):2425. doi: 10.3390/nu17152425 (PMC12348034; doi:10.3390/nu17152425)
Supplement: Supplementary file 1 [file nutrients-17-02425-s001.zip › nutrients-3750826-supplementary.pdf]

# Supplementary Materials

Table S1: Normality test results and corresponding statistical test selection for each band–channel pair.

| Band | Channel | Shapiro–Wilk $p$-value | Normally Distributed | Test Used |
|---|---|---|---|---|
| All (1–45 Hz) | Cz | 0.00086 | No | wilcoxon |
| All (1–45 Hz) | F7 | 0.04738 | No | wilcoxon |
| All (1–45 Hz) | Fp2 | 0.83243 | Yes | paired t-test |
| All (1–45 Hz) | Fz | 0.93386 | Yes | paired t-test |
| All (1–45 Hz) | T4 | 0.78116 | Yes | paired t-test |
| All (1–45 Hz) | T6 | 0.43238 | Yes | paired t-test |
| Alpha (7–12 Hz) | P4 | 0.06749 | Yes | paired t-test |
| Beta (12–30 Hz) | T4 | 0.67298 | Yes | paired t-test |
| Beta (12–30 Hz) | T6 | 0.32096 | Yes | paired t-test |
| Delta (1–3 Hz) | Cz | 0.01922 | No | wilcoxon |
| Delta (1–3 Hz) | Fp2 | 0.71432 | Yes | paired t-test |
| Delta (1–3 Hz) | FpZ | 0.57738 | Yes | paired t-test |
| Delta (1–3 Hz) | Fz | 0.90607 | Yes | paired t-test |
| Delta (1–3 Hz) | OZ | 0.23341 | Yes | paired t-test |
| Delta (1–3 Hz) | Pz | 0.00508 | No | wilcoxon |
| Gamma (30–45 Hz) | Cz | 0.00049 | No | wilcoxon |
| Gamma (30–45 Hz) | F7 | 0.32711 | Yes | paired t-test |
| Gamma (30–45 Hz) | Fz | 0.75899 | Yes | paired t-test |
| Gamma (30–45 Hz) | Pz | 0.00118 | No | wilcoxon |
| Gamma (30–45 Hz) | T4 | 0.52897 | Yes | paired t-test |
| Gamma (30–45 Hz) | T5 | 0.00098 | No | wilcoxon |
| Gamma (30–45 Hz) | T6 | 0.50454 | Yes | paired t-test |
| Theta (3–7 Hz) | Cz | 0.00573 | No | wilcoxon |
| Theta (3–7 Hz) | Fp2 | 0.31220 | Yes | paired t-test |

Table S2: Statistical analyses evaluating sex-related effects in EEG features with significant pre–post differences. Features include frequency band powers, ratios, or band–channel combinations. Normality was assessed using the Shapiro–Wilk test. Depending on distribution, either an independent samples t-test or Mann–Whitney U test was used. Effect sizes (Cohen's $d$), participant counts ($N_F$, $N_M$), and FDR-adjusted $q$-values are provided. NaN (Not a Number) indicates no t-value due to non-parametric test.

| **Features** | **Normality *p*** | **Test** | ***p*-value** | **t / NaN** | **Cohen's *d* / r** | $N_F$ | $N_M$ | ***q*-value** |
|---|---|---|---|---|---|---|---|---|
| Total Power | 0.102 | Independent samples t-test | 0.401 | -0.861 | -0.393 | 8 | 12 | 0.913 |
| Delta Power | 0.466 | Independent samples t-test | 0.491 | -0.702 | -0.321 | 8 | 12 | 0.913 |
| Theta Power | 0.3 | Independent samples t-test | 0.445 | -0.781 | -0.357 | 8 | 12 | 0.913 |
| Alpha Power | 0.136 | Independent samples t-test | 0.859 | -0.180 | -0.082 | 8 | 12 | 0.913 |
| Beta Power | 0.039 | Mann–Whitney U test | 0.616 | Nan | -0.367 | 8 | 12 | 0.913 |
| Gamma Power | 0.018 | Mann–Whitney U test | 0.671 | Nan | -0.355 | 8 | 12 | 0.913 |
| Relative Delta | 0.005 | Mann–Whitney U test | 0.847 | Nan | 0.074 | 8 | 12 | 0.913 |
| Relative Theta | 0.004 | Mann–Whitney U test | 1.000 | Nan | -0.019 | 8 | 12 | 1.000 |
| Relative Alpha | 0.009 | Mann–Whitney U test | 0.671 | Nan | 0.113 | 8 | 12 | 0.913 |
| Relative Beta | 0.006 | Mann–Whitney U test | 0.335 | Nan | 0.551 | 8 | 12 | 0.913 |
| Relative Gamma | 0.004 | Mann–Whitney U test | 0.847 | Nan | -0.160 | 8 | 12 | 0.913 |
| FAA | 0.058 | Independent samples t-test | 0.129 | 1.608 | 0.787 | 8 | 12 | 0.913 |
| Alpha Beta | 0.046 | Mann–Whitney U test | 0.787 | Nan | -0.158 | 8 | 12 | 0.913 |
| Delta Alpha | 0.014 | Mann–Whitney U test | 0.847 | Nan | 0.212 | 8 | 12 | 0.913 |
| Alpha Theta | 0.394 | Independent samples t-test | 0.346 | 0.967 | 0.441 | 8 | 12 | 0.913 |
| Theta Beta | 0.001 | Mann–Whitney U test | 0.616 | Nan | -0.259 | 8 | 12 | 0.913 |
| Beta Gamma | 0.064 | Independent samples t-test | 0.185 | 1.378 | 0.629 | 8 | 12 | 0.913 |
| All - Cz | 0.033 | Mann–Whitney U test | 0.671 | Nan | -0.147 | 8 | 12 | 0.866 |
| All - F7 | 0.023 | Mann–Whitney U test | 0.238 | Nan | -0.676 | 8 | 11 | 0.837 |
| All - Fp2 | 0.185 | Independent samples t-test | 0.305 | 1.059 | 0.512 | 7 | 11 | 0.837 |
| All - Fz | 0.311 | Independent samples t-test | 0.314 | -1.036 | -0.473 | 8 | 12 | 0.837 |
| All - T4 | 0.314 | Independent samples t-test | 0.792 | 0.269 | 0.147 | 5 | 10 | 0.888 |
| All - T6 | 0.126 | Independent samples t-test | 0.446 | 0.782 | 0.386 | 7 | 10 | 0.866 |
| Alpha - P4 | 0.022 | Mann–Whitney U test | 0.740 | Nan | -0.023 | 7 | 10 | 0.866 |
| Beta - T4 | 0.324 | Independent samples t-test | 0.548 | 0.617 | 0.338 | 5 | 10 | 0.866 |
| Beta - T6 | 0.113 | Independent samples t-test | 0.453 | 0.770 | 0.379 | 7 | 10 | 0.866 |
| Delta - Cz | 0.222 | Independent samples t-test | 0.569 | -0.581 | -0.265 | 8 | 12 | 0.866 |
| Delta - Fp2 | 0.1 | Independent samples t-test | 0.518 | 0.662 | 0.320 | 7 | 11 | 0.866 |
| Delta - FpZ | 0.905 | Independent samples t-test | 0.965 | -0.045 | -0.027 | 4 | 9 | 0.984 |
| Delta - Fz | 0.115 | Independent samples t-test | 0.872 | -0.164 | -0.075 | 8 | 12 | 0.918 |
| Delta - OZ | 0.286 | Independent samples t-test | 0.725 | -0.358 | -0.170 | 8 | 10 | 0.866 |
| Delta - Pz | 0.061 | Independent samples t-test | 0.131 | 1.584 | 0.723 | 8 | 12 | 0.837 |
| Gamma - Cz | 0.018 | Mann–Whitney U test | 0.847 | Nan | -0.034 | 8 | 12 | 0.908 |
| Gamma - F7 | 0.230 | Independent samples t-test | 0.147 | -1.519 | -0.706 | 8 | 11 | 0.837 |
| Gamma - Fz | 0.181 | Independent samples t-test | 0.266 | -1.149 | -0.524 | 8 | 12 | 0.837 |
| Gamma - Pz | 0.004 | Mann–Whitney U test | 0.083 | Nan | 0.879 | 8 | 12 | 0.837 |
| Gamma - T4 | 0.233 | Independent samples t-test | 0.716 | 0.372 | 0.204 | 5 | 10 | 0.866 |
| Gamma - T5 | 0.002 | Mann–Whitney U test | 0.301 | Nan | -0.814 | 6 | 11 | 0.837 |
| Gamma - T6 | 0.765 | Independent samples t-test | 0.355 | 0.955 | 0.471 | 7 | 10 | 0.849 |
| Theta - Cz | 0.042 | Mann–Whitney U test | 0.375 | Nan | -0.239 | 8 | 12 | 0.849 |
| Theta - Fp2 | 0.119 | Independent samples t-test | 0.597 | 0.539 | 0.261 | 7 | 11 | 0.866 |
